# Differential Integration of Transcriptome and Proteome Identifies Pan-Cancer Prognostic Biomarkers

**DOI:** 10.3389/fgene.2018.00205

**Published:** 2018-06-15

**Authors:** Gregory W. Schwartz, Jelena Petrovic, Yeqiao Zhou, Robert B. Faryabi

**Affiliations:** ^1^Department of Pathology and Laboratory Medicine, Perelman School of Medicine at the University of Pennsylvania, Philadelphia, PA, United States; ^2^Abramson Family Cancer Research Institute, Perelman School of Medicine at the University of Pennsylvania, Philadelphia, PA, United States; ^3^Institute for Biomedical Informatics, Perelman School of Medicine at the University of Pennsylvania, Philadelphia, PA, United States

**Keywords:** data integration, network analysis, proteomics, transcriptomics, cancer biology

## Abstract

High-throughput analysis of the transcriptome and proteome individually are used to interrogate complex oncogenic processes in cancer. However, an outstanding challenge is how to combine these complementary, yet partially disparate data sources to accurately identify tumor-specific gene products and clinical biomarkers. Here, we introduce inteGREAT for robust and scalable differential integration of high-throughput measurements. With inteGREAT, each data source is represented as a co-expression network, which is analyzed to characterize the local and global structure of each node across networks. inteGREAT scores the degree by which the topology of each gene in both transcriptome and proteome networks are conserved within a tumor type, yet different from other normal or malignant cells. We demonstrated the high performance of inteGREAT based on several analyses: deconvolving synthetic networks, rediscovering known diagnostic biomarkers, establishing relationships between tumor lineages, and elucidating putative prognostic biomarkers which we experimentally validated. Furthermore, we introduce the application of a clumpiness measure to quantitatively describe tumor lineage similarity. Together, inteGREAT not only infers functional and clinical insights from the integration of transcriptomic and proteomic data sources in cancer, but also can be readily applied to other heterogeneous high-throughput data sources. inteGREAT is open source and available to download from https://github.com/faryabib/inteGREAT.

## 1. Introduction

Cellular processes are tightly regulated in multiple layers, leading to coordinated function of genes and gene products including transcripts and proteins. Aberrations at each tier of these multilayer regulatory circuits could lead to malignant transformations. It has been shown that combined analysis of data characterizing a variety of biomolecules yields discovery of new insights into tumor biology and facilitates identification of important cancer genes and therapeutic targets (Chang et al., 2013; Zhang et al., 2014; Mertins et al., 2016; Zhang et al., 2016). These initiatives have increased interest in development of methods for integration of heterogenous data sources (Huang et al., 2013; Meng et al., 2016; Petralia et al., 2016).

The interrogation of information garnered by high-throughput measurements of transcripts or proteins have been used to refine stratification of tumors based on their unique molecular characteristics. Furthermore, analysis of each of these data sources separately has facilitated the discovery of transcript- or protein-based prognostic and diagnostic biomarkers. The salient assumption underlying such comparative studies is that there is a one-to-one relationship between transcript and protein expression, however previous studies have shown low correlation between these levels (Haider and Pal, 2013; Zhang et al., 2014). Another implicit assumption is that genome-scale technologies such as next generation sequencing-based transcriptomics and mass spectrometry-based proteomics have comparable sensitivity to capture the activities of these gene products. Yet, examining each aspect of tumor pathobiology alone overlooks potential regulatory mechanisms relating the gene products and the technologies measuring these aspects do not have the same coverage. It is therefore critical to effectively combine information gathered by complementary genome-scale measurements to elucidate common and different molecular features of tumor types.

To address this challenge, methods to integrate heterogeneous data sources such as transcriptomic and proteomic data sets have been proposed (Haider and Pal, 2013). These integration methods range from naive weighted means of transcript and protein abundances (Balbin et al., 2013) to consensus pathways and molecules (Wachter and Beißbarth, 2016). Other approaches take advantage of the relationships between gene products to produce a network of associated genes, known as an interactome (Gibbs et al., 2014). *De novo* clustering of interactomes was used to elucidate a subnetwork or pathway containing gene products with functional relatedness (Dutkowski et al., 2012). Some techniques instead integrate data sources before clustering using a joint latent model with some success (Shen et al., 2009; Michaut et al., 2016). Summarizing the information within each cluster using eigenvectors provides a means to compare clusters (Gibbs et al., 2014). Measuring the network structure between different levels was also proposed as a means for data integration (Cho et al., 2016). The disadvantage of grouping gene products is that collapsing these structures into clusters can decrease the sensitivity of biomarker detection. For instance, while a cluster may be classified as clinically significant, an important gene may belong to a different cluster depending on the clustering parameters and algorithm. Grouping gene products also complicates devising gene-centric biomarkers that are the main focus of diagnostic tests. Merging networks to create a summary network was also proposed to address the shortcomings of the clustering-based approaches (Franceschini et al., 2012; Wong et al., 2015). These methods of integration were performed on a single phenotype and thus cannot readily identify phenotypic biomarkers differentiating tumor subtypes. We propose that expanding differential expression analysis from the individual level to differential integration can facilitate biomarker discovery.

To this end, we present inteGREAT, an algorithm for differential integration. inteGREAT generates interactomes for both transcriptomes and proteomes and analyzes their network structures to determine the extent by which a gene product and its related partners are similar across different sources of data while different between cellular phenotypes. Using a framework based on both “local” and “global” similarity, inteGREAT provides a robust and scalable algorithm that can integrate any number of genomic and functional genomic data sets to identify differentiating tumor biomarkers. inteGREAT by design does not cluster gene products at any point in order to retain individual relationships and is thus able to assign confidence of integration to each gene representing its transcript and protein expressions. We assessed the ability of inteGREAT to detect perturbations in multiple networks through simulations. Using breast cancer transcriptome and proteome data from The Cancer Genome Atlas (TCGA) (Grossman et al., 2016) and the Clinical Proteomic Tumor Analysis Consortium (CPTAC) (Ellis et al., 2013; Edwards et al., 2015), we demonstrated the utility of inteGREAT to identify subtype-specific biomarkers in breast cancer. inteGREAT is a robust, easy to use software package and can be generally applied to any abundance data or pre-made network. inteGREAT is open source and available to download from https://github.com/faryabib/inteGREAT.

We further applied inteGREAT in a pan-cancer integrative analysis of transcriptome and proteome data sets from TCGA and CPTAC for serous ovarian carcinoma (OV) (Bell et al., 2011; Zhang et al., 2016), breast cancers (BRCA) (Koboldt et al., 2012; Mertins et al., 2016), colon (COAD), and rectal (READ) adenocarcinomas (Muzny et al., 2012; Zhang et al., 2014). We proposed using a measure of clumpiness on the resulting hierarchy of comparisons that elucidated the promiscuous nature of the luminal and HER2-positive subtypes, while demonstrating the relative isolation of ovarian, colorectal, and to some extent basal subtypes. Our integrative pan-cancer analysis quantitates the importance of each individual gene in stratifying a particular subtype. Among them, we identified a set of clinically important genes that are strongly associated with prognostic outcomes in a given tumor type. Our differential integration of transcript and protein abundance across four tumor types is a showcase of using inteGREAT for similar integration analysis in other cancers and diseases.

## 2. Materials and methods

### 2.1. inteGREAT algorithm overview

**Figure 1:**
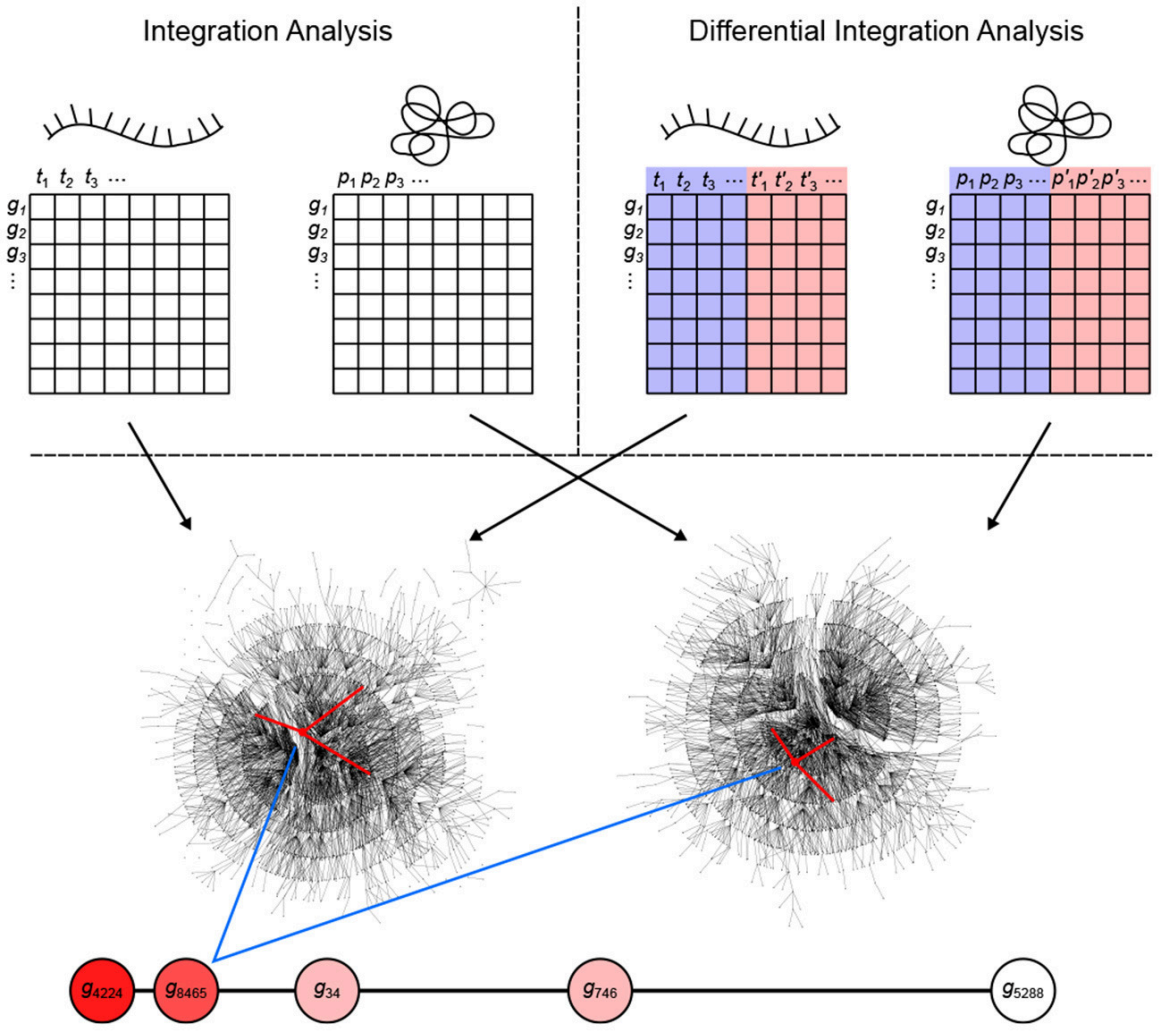
Overview of the inteGREAT algorithm. inteGREAT supports both a non-differential (top left) and a differential (top right) integration analysis. Abundance data for all genes from the transcriptome (left matrix for both analyses) and proteome (right matrix for both analyses) provides information to generate a correlation network for each data source. For the differential integration analysis, these abundance values comes from two phenotypes as shown by the red and blue overlays. inteGREAT measures the local (structure of the immediate neighbors, one hop away, shown by red-marked edges) or global similarity (structure of the immediate neighborhood, multiple hops away as defined by the random walk with restart) for each gene and produces a ranked-order list of putative biomarkers with assigned confidences. Increasing red fills of nodes mark ascending gene rank.

inteGREAT is an algorithm for integration of disparate high-throughput data sets. This algorithm can also perform differential integration for comparative analyses of multiple cellular phenotypes. Differential integration is crucial to stratify two phenotypes and uncover genes leading to molecular differences between tumors. inteGREAT achieves differential integration in three stages: network generation, network similarity, and vertex joining (Figure 1). inteGREAT first creates two undirected weighted graphs of correlations between gene products for the transcriptome and proteome, called interactomes, separately. In the transcript interactome each vertex represents a gene's transcript, while in the proteome interactome each vertex represents the protein product of that gene. Each vertex maps to a vector of abundances, where each index is the abundance of that gene product in a sample. For differential integration, these samples come from two different phenotypes. The edge weight between two gene products is set to the correlation between their abundance vectors.

To determine vertex-wise network similarity, inteGREAT provides two methods. First, inteGREAT analyzes the structure of the immediate neighborhood of a gene in each interactome and calculates the cosine similarity between gene products. In this method, a vector is assigned to each interactome vertex consisting of the edge weights of its immediate neighbors per interactome. Second, for an expanded measure of topology, inteGREAT uses random walk with restart to obtain a stationary distribution centered around a gene product in both interactomes and then determines the concordance in structure using cosine similarity between the two distributions. A random walk with restart provides a more global view of the vertex neighborhood (global similarity), while cosine similarity efficiently compares a vertex's immediate neighbors across two measurement levels (local similarity). By analyzing the topological structure of each interactome before collapsing protein and transcript measurements into a single gene identifier, we can observe relationships of gene products at each level without loss of information. The value resulting from the network similarity step represents how conserved the interaction neighborhood of a gene product is between the interactomes generated from the transcriptome and proteome assays. The joining step produces a final result as a ranked-order of gene product cosine similarities. A differential integration using both tumor types reinterprets this value as conserved behavior across assays but different between the two cellular phenotypes.

### 2.2. Correlation network generation

To integrate measurements of *l* distinct data sources such as transcriptome and proteome, inteGREAT first generates the undirected weighted graph of correlation networks for each data source μ ∈ {1, …, *l*} separately, where each gene product is a vertex and each edge weight is a correlation between two adjacent vertices based on their abundance values in the data source μ. Let *G* be the set of gene products. inteGREAT creates the data source μ correlation network adjacency matrix **A**_μ_, where for each gene product pair *i, j* ∈ *G*

(1) $$\begin{array}{l} {\text{A}}_{\mu }\left[i,j\right]=\{\begin{array}{cc} \rho_{\mu },\; & p_{\mu }<0.05\\ 0,\; & \text{otherwise} \end{array} \end{array}$$

such that ρ_μ_ and *p*_μ_ are correlation coefficients and *p*-values respectively. The correlation coefficient is calculated between two abundance vectors in the data source μ. The abundance vector of a gene product *i* is defined as 〈*g*_μ_(*i*, 1), …, *g*_μ_(*i, m*), *g*_μ_(*i, m* + 1), …, *g*_μ_(*i, n*)〉 for differential integration where *g*_μ_(*i, k*) denotes the abundance of gene product *i* from sample *k* of the data source μ. Here, there are *m* samples from one phenotype and *n*−*m* samples from another phenotype. All samples are from one phenotype in the case of non-differential integration analysis. inteGREAT implements several options for correlation measures, such as Pearson (Pearson, 1895) and Spearman (Spearman, 1904). The latter measure is a rank transformation of the former, so the resulting correlation network may vary significantly between the two methods. inteGREAT can take as input either normalized abundance data and generate these networks or accept pre-made networks.

### 2.3. Vertex similarity calculation

After generating the correlation network adjacency matrices from the abundance measurements of each data source, inteGREAT relates the vertices of each pair of adjacency matrices **A**_μ_ and **A**_ν_ by calculating a “vertex similarity” score vector **c**_μ, ν_ = 〈*c*_μ,ν_[1], …, *c*_μ,ν_[|*G*|]〉, for each pair of **A**_μ_ and **A**_ν_ in the set of network adjacency matrices *U* = {**A**_1_, **A**_2_, …, **A**_*l*_}. Here, *c*_μ,ν_[*i*] is the similarity of gene product *i* between two adjacency matrices **A**_μ_ and **A**_ν_. inteGREAT implements two distinct measures to calculated vertex similarity scores: “local similarity” and “global similarity.” The local similarity considers the network topology only one hop away from a vertex by looking only at the edges at that vertex (Figure 1, red-marked edges). Let the cosine similarity between two vectors, **x** and **y**, of equal length be defined as (Salton et al., 1975)

(2) $$\begin{array}{l} C\left(\text{x},\text{ y}\right)=\frac{\sum_{i=1}^{\left|G\right|}\text{x}\left[i\right]\text{y}\left[i\right]}{\sqrt{\sum_{i=1}^{\left|G\right|}\text{x}{\left[i\right]}^{2}}\sqrt{\sum_{i=1}^{\left|G\right|}\text{y}{\left[i\right]}^{2}}}; \end{array}$$

then the *local* similarity defines the score vector as

(3) $$\begin{array}{l} {\text{c}}_{\mu ,\nu }\left[i\right]=C\left({\text{A}}_{\mu }\left[i,\cdot \right],{\text{A}}_{\nu }\left[i,\cdot \right]\right) \end{array}$$

is the local similarity score of gene product *i* between **A**_μ_ and **A**_ν_.

Alternatively, inteGREAT determines the global similarity by examining the expanded topology of the network from each vertex using a random walk with restart (Leiserson et al., 2014). In this case, the neighborhood structure of a vertex takes into account multiple hops away from the vertex instead of only the immediate neighbors, including any loops within the network. The *global* similarity defines the score vector as

(4) $$\begin{array}{l} {\text{c}}_{\mu ,\nu }\left[i\right]=C\left({\text{s}}_{\mu }\left[i,\cdot \right],{\text{s}}_{\nu }\left[i,\cdot \right]\right), \end{array}$$

where **s**_μ_[*i*, ·] and **s**_ν_[*i*, ·] are the stationary distributions for transitioning from gene product *i* to any other gene product in **A**_μ_ and **A**_ν_, respectively. The stationary distribution of gene product *i* in a network with adjacency matrix **A**_*z*_ with restart is defined as

(5) $$\begin{array}{l} {\text{s}}_{z}\left[i,\cdot \right]=\beta_{i}{\left(\text{I}-\left(1-\beta_{i}\right){\text{W}}_{z}\right)}^{-1}{\text{e}}_{z}\left(i\right), \end{array}$$

where **I** is the identity matrix, **e**_*z*_(*i*) contains 1 at position *i* and 0 elsewhere, β_*i*_ is the restart probability at vertex *i*, and **W**_*z*_[*i, j*] is the probability of traveling from *i* to *j* (Leiserson et al., 2014). **W**_*z*_ is calculated from **A**_*z*_, such that $\sum_{j=1}^{\left|G\right|}{\text{W}}_{z}\left[i,j\right]=1$, ∀*i, j* ∈ {1, 2, …, |*G*|}, and **W**_*z*_[*i, j*] ∈ [0, 1].

### 2.4. Vertex joining

For each gene product *i*, inteGREAT calculates the final similarity score **c**[*i*] by joining the **c**_μ,ν_[*i*] calculated for each pair of network adjacency matrices in *U* = {**A**_1_, **A**_2_, …, **A**_*l*_}. For *l* > 2, a joining function *f* combines the calculated **c**_μ,ν_[*i*] into the final similarity score of gene product *i* as **c**[*i*] = *f*(**c**_1,2_[*i*], …, **c**_*l*−1,*l*_[*i*]) from all the pairwise similarity scores **c**_μ,ν_[*i*]. For the function *f*, inteGREAT defaults to the arithmetic mean but one can use other options including the maximum, minimum, and geometric mean. In the case of integrating only two data sources such as transcriptomics and proteomics, *f* is the identity function. In our simulation study with 3 data sources, we used the inteGREAT default *f* and calculated the similarity score of gene product *i* as **c**[*i*] = *f*(**c**_1,2_[*i*], **c**_1,3_[*i*], **c**_2,3_[*i*]).

We then assign confidence intervals to each final similarity score **c**[*i*], for *i* ∈ {1, …, |*G*|} using the bias-corrected and accelerated (BCa) bootstrap (Efron, 1987). As cosine similarity is the last step to calculate both the global and local similarity scores **c**[*i*], we can use bootstrapping on the cosine similarity between two vectors at this same step. Let **x** and **y** be two vectors of length |*G*|. Then let α and β be two vectors of length *n* < |*G*|, such that α[*i*] = **x**[*j*] and β[*i*] = **y**[*j*] ∀*i* ∈ {1, 2, …, |*G*|}∧∀*j* ∈ {1, 2, …, *n*} (so the relationship of indices are maintained from **x** and **y** to α and β). Our bootstrapping function is then *C*(α, β). We expect the resampled vectors to have a similar direction as the complete vectors. From this analysis we obtain the confidence interval as well as the confidence interval width, the measure we use to assign confidence.

## 3. Results

### 3.1. inteGREAT provides robust measures of inter-network similarity

A key component of differential integration is detecting changes concordantly reflected across multiple gene products associated with a given gene. However, technical or experimental variabilities can lead to noisy high-throughput data sets, potentially resulting in unreliable inference. Missing or inaccurate gene product readouts and variability in the assays can result in interaction networks with missing vertices, misplaced edges, and noisy edge values. As inteGREAT detects changes concordant across different data sources using network similarity, we evaluated how unreliable data impacts identifying similarity between networks.

**Figure 2:**
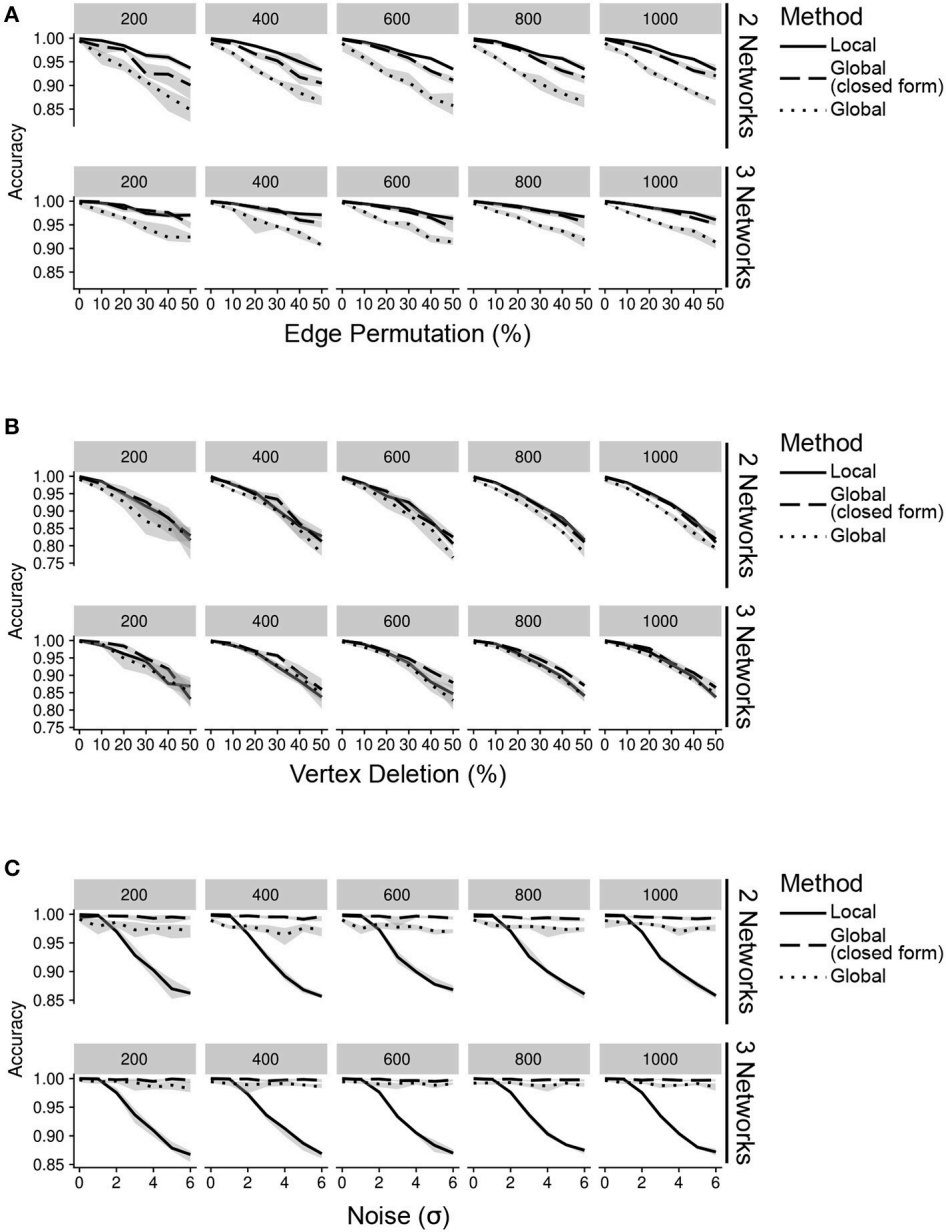
Accuracy of inteGREAT for various noise types across different number of data sources (networks) and network sizes (facet label). **(A)** Simulations with varying percent of the edges permuted. Existing edges are randomly chosen for permuting based on a uniform distribution. **(B)** Simulations with varying percent of the vertices deleted. Vertices are randomly chosen for deletion using a uniform distribution. **(C)** Simulations with varying amount of noise. Noise refers to the addition of random values to the network edges drawn from a Gaussian distribution with a standard deviation of the x-axis.

In order to mimic a biological network with hubs, we randomly generated a network using the Barabási-Albert model (Barabási and Albert, 1999) to represent an interaction network from a single level of data. In the absence of noise, this network represents a single interactome produced by any of the data sources. To simulate interactomes from additional data sources, we generated a new network by permuting 5% of the vertices in the original network. These vertices were the “difference” between data sources and acted as known changes inteGREAT attempted to detect. We simulated the scenarios when two or three data sources are available, such as transcriptomics, proteomics, and phosphoproteomics and assessed the performance of inteGREAT with global or local similarity (Figure 2, see Supplementary Materials). To complement the stationary distribution of the random walk with global similarity, we included the result of having simulated random walk transition through the network with restart. As the result of inteGREAT is a ranked-order of genes, we measured accuracy by the overall distance of each changed vertex from its expected location at the end of the list (Table S1, see Supplementary Materials).

We first simulated scenarios where noisy measurements result in false relationships between gene products. To this end, we permuted 0–50% of edges in the network. This permutation could model differences between the molecular species measured by each high-throughput technology. Regardless of the similarity measure, inteGREAT was invariant to the size of the network but became slightly more accurate when the behavior of a cellular system was characterized with three interactomes (Figure 2A), suggesting a potential benefit in investigating a phenotype at multiple data sources—for instance, including not just the transcriptome or the proteome, but the epigenome as well. We also observed similar accuracy for both local and global similarity. inteGREAT with local similarity exhibited slightly improved performance when two data sources were considered, suggesting that measuring direct neighbors instead of an interactome global view captured by random walk is more robust when two gene products are associated based on one data source but are independent based on the other vantage point of the system.

We next sought to explore the effect of missing information on our network similarity measures through vertex deletion (Figure 2B). Missing data is common when comparing transcriptome and proteome measurements of the same cellular condition, as the breadth (number of measured proteins) may not encompass all the genes found in the transcriptome analysis. To simulate missing data, we randomly deleted 0–50% of the vertices. Among the simulated scenarios, vertex deletion resulted in the worst performance compared to the other sources of noise, suggesting the lack of measurement of a gene product's neighbor in a data source could not be fully compensated for by observing that neighbor at alternative levels. Integrating data sources from a number of high-throughput technologies with different breadth in measurements significantly limits the accuracy of the integration analyses. Comparison of integration analyses when two or three data sources were available showed that an additional data source enhanced the accuracy of the integration analysis, implying that more comprehensive characterizations of a cellular phenotype using complementary assays could alleviate the detrimental effect of imbalanced breadths of various technologies and missing data.

We also simulated the effect of noisy high-throughput experiments on the accuracy of transcriptomics and proteomics integrative analysis. To simulate this source of network inaccuracy, we injected noise into each edge from a normal distribution with σ from 0 to 6 (Figure 2C). This simulation resulted in a striking difference between the inteGREAT performance with local and global similarity. The performance of inteGREAT with global similarity was minimally impacted by the introduced noise, while inteGREAT with local similarity exhibited performance decrease proportional to the noise level. (Figure 2C). Nevertheless, inteGREAT performed with >0.8 accuracy, which is significantly higher than the worst-case accuracy of 0.5 resulting from changed vertices uniformly distributed among the ranked-order list. This result suggests that inteGREAT can be reliably deployed even in the presence of some degree of inconsistency between networks and is robust to noisy measurements.

### 3.2. inteGREAT rediscovers canonical biomarkers of breast cancer subtypes

Although integration of synthetic networks demonstrated the robustness of inteGREAT in the presence of various sources of noise in the measurements, simulated data are generally limited in recapitulating the complexity of real biological data sets. To further validate inteGREAT's performance in a biological setting, we next investigated the ability of inteGREAT to identify biomarkers associated with a given cellular phenotype from the integration of transcriptomic and proteomic data sets.

**Figure 3:**
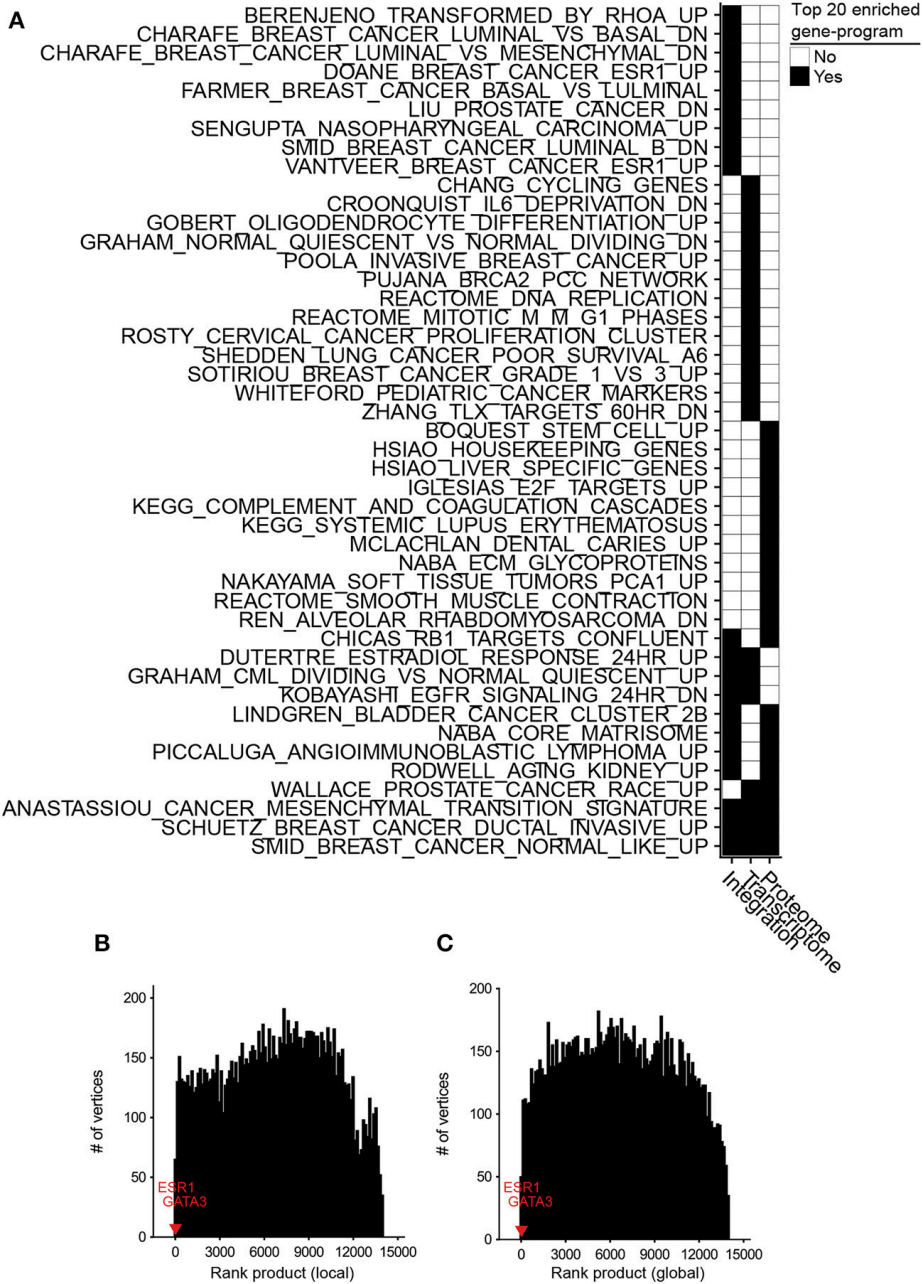
Differential integration of basal vs. luminal breast cancer subtypes identified known gene-programs associated with these tumor subtypes and detected *ESR1* and *GATA3* as their differential biomarkers. **(A)** Top 20 gene-programs associated with the most differential genes between basal and luminal breast cancer subtypes for three differential analyses: integration of transcriptome and proteome, transcriptome only, and proteome only. Pre-ranked GSEA analysis was performed based on the gene-programs defined by MSigDB C2 curated gene sets and the ranked-order list of differential genes generated by each analysis. Black cells signify a gene set or pathway in the top 20 most significantly enriched pathways for that column. **(B)** Ten runs of differential integration of basal vs. luminal using local similarity. The final ranked-order list was generated from the joining of each ranked order-lists using the rank product. *ESR1* and *GATA3* are marked with red and blue respectively. **(C)** Final rank product of 10 runs based on global similarity.

We conducted differential integrative analyses using TCGA transcriptomic and CPTAC proteomic data sets (Table S2) of basal and luminal breast cancer subtypes (Farmer et al., 2005). The inteGREAT differential integration analysis resulted in a ranked-order list of 13,958 gene identifiers (representing respective gene products) from the most to the least conserved between the transcriptome and proteome and differential between the basal and luminal subtypes. We hypothesized that the genes with the most conserved neighborhoods in all data sources and different between the luminal and basal subtypes would be placed at the top of the ranked-order list. To test this hypothesis, we benefited from the curated MSigDB gene set database (Liberzon et al., 2011) and performed unbiased gene set enrichment analyses (GSEA) (Subramanian et al., 2005) on the ranked-order list outputted by the inteGREAT differential integration analysis. The genes identified by inteGREAT as highly different between the luminal and basal subtypes while exhibiting concordant transcript and protein neighborhood topologies were significantly enriched with the gene-programs known to differentiate basal and luminal subtypes (Figure 3A, Table S3). These sets included genes that are positively regulated by estrogen receptor ERα, genes upregulated after estradiol treatment, and genes reported as differential biomarkers of luminal versus basal subtypes in two independent studies (Figure 3A, Table S3). Conversely, the genes that were ranked low and uncorrelated between the data sources were overrepresented in more general pathways unrelated to the pathobiology of basal, luminal, or breast cancer such as HIV infection or proteasomes (Table S4). This result suggests that inteGREAT correctly identified the gene-programs and pathways discriminating between these two breast cancer subtypes from the ones that are irrelevant to this comparative study.

We also assessed the benefit of integrating transcriptomic and proteomic data sources rather than using only one source by comparing the results of integrative and single data source analyses. To this end, we applied inteGREAT such that the two interactomes were generated from only the basal or luminal transcriptomic data sets, and used inteGREAT to identify the differences between the two transcriptomic networks. We also performed a similar single data source analysis based on the proteomic data sets instead of transcriptomic measurements. These two analyses resulted in two ranked-order lists of genes: one from the transcriptome and the other from the proteome. Compared to the differential integrative analysis, single data source analysis based on the transcriptome or the proteome alone both detected fewer gene sets implicated in the pathobiology of breast cancer and the differences between the basal and luminal subtypes (Figure 3A, Tables S5, S6). For instance, neither of the single data source analysis were able to identify the genes positively regulated by *ESR1*, a known activated pathway in the luminal subtype. Together, these analyses exhibit the ability of inteGREAT differential integration analysis to not only elucidate some of the known gene-programs and pathways associated with the differences between the basal and luminal subtypes, but also underscores the benefit of additional data sources for more accurate integrative analysis.

One of the advantages of not collapsing genes to pathways is the direct identification of potential biomarkers of a tumor subtype. *ESR1* and *GATA3* are reported as differential biomarkers of basal and luminal subtypes (Farmer et al., 2005; Chou et al., 2010; Jiang et al., 2014). In the ranked list of 13,958 gene identifiers resulted by the differential integration analysis of basal vs. luminal with local similarity, *ESR1* and *GATA3* were ranked 2nd (CI: 0.377–0.407) and 7th (CI: 0.356–0.390), respectively (Table S7). We compared these rankings to integration analysis within each single tumor subtype to assess the benefit of biomarker detection using differential integration. Without differential integration, we observed a marked decrease in the rankings for these known differential biomarkers of breast cancer subtypes. Integration of basal subtype proteomic and transcriptomic data ranked *ESR1* and *GATA3* at 8,889 (CI: 0.0100–0.0391) and 2,754 (CI: 0.0365–0.0705), respectively. Integrative analysis of luminal A subtype ranked *ESR1* and *GATA3* at 1,688 (CI: 0.0558–0.0915) and 9,027 (CI: 0.0166–0.0483), respectively. While the similar analysis in luminal B, resulted in 133 (CI: 0.181–0.220) and 2,345 (CI: 0.0607–0.0990) ranking of *ESR1* and *GATA3*, respectively. Orthogonal to the integrative analysis within a single tissue, we looked at the differential between basal and luminal using local similarity from a single data source (Figures S1A,B). Here, *ESR1* and *GATA3* ranked 7,641 (CI: 0.0244–0.0575) and 7,076 (CI: 0.0322–0.0688) in the transcriptome and 8,898 (CI: 0.0123–0.0527) and 8,482 (CI: 0.0170–0.0612) in the proteome, respectively. Furthermore, typical differential fold change analysis of each data source ranked *ESR1* and *GATA3* at 5 and 128 most differential transcripts, respectively. Similar analysis of proteome data set ranked *ESR1* and *GATA3* as 17 and 23 most differential proteins between the luminal and basal subtypes (Figures S1C,D). Together this analysis demonstrates that nominating potential biomarkers by differential integration is in the orders of magnitude more accurate than the integration analysis of each tumor subtype separately and outperforms typical differential fold change analysis, pointing to the benefits of a differential integration analysis (Table S7).

Potential biases in the collected data sources could adversely impact analyzing the interactome structures separately. For instance, it is common to have more samples from one assay over another. In the TCGA/CPTAC breast cancer data sets, there are 29 more proteome samples compared to transcriptome samples. As a result, there could be some degree of overfitting in a co-expression network construction leading to more accurate inference of the network with a larger sample size. Hence, we postulated that enrichment of proteome samples might have resulted in a bias in our networks. In order to evaluate the robustness of inteGREAT to uneven number of samples between the two data sources, we randomly sub-sampled our basal and luminal data sets such that there were an equal number of samples for the transcriptome and the proteome. The similarity and the confidence interval (CI) width was calculated with local (representative run, Figures S2A,C) or global similarity (representative run, Figures S2B,D) for each sub-sampled set. The aggregate ranking of the genes was calculated by combining the results of 10 sub-sampled sets using rank product with 1,000 permutations (Figures 3B,C). inteGREAT with local similarity ranked *ESR1* and *GATA3* as the 2nd (*p* < 1e-16) and 16th (*p* < 1e-16) most conserved gene products that are at the same time differential between basal vs. luminal subtypes (Figure 3B and Table S8), respectively. A similar analysis using inteGREAT with global similarity ranked *ESR1* 3rd (*p* < 1e-16) and *GATA3* 22nd (*p* < 1e-16) (Figure 3C and Table S9). These results corroborate with the expected biomarkers shown to differentiate these two breast cancer subtypes (Farmer et al., 2005; Chou et al., 2010; Jiang et al., 2014), implying the robustness of our framework to biased sample sizes.

### 3.3. inteGREAT relates molecular signatures and tissue-of-origin tumor classification

After establishing the ability of inteGREAT to identify differential biomarkers of basal versus luminal breast cancer subtypes, we sought to use inteGREAT to elucidate relationships between molecular underpinnings of cancer types and their site-of-origin, and benchmarked our results against (Hoadley et al., 2014) to assess the inteGREAT performance. To this end, we expanded our data set to encompass transcriptomic and proteomic data sets for serous ovarian carcinoma (OV) (Bell et al., 2011; Zhang et al., 2016), breast cancers (BRCA) (Koboldt et al., 2012; Mertins et al., 2016), colon (COAD), and rectal (READ) adenocarcinomas (Muzny et al., 2012; Zhang et al., 2014) (Table S2, see Supplementary Materials). We applied inteGREAT to each of the malignancies in our data set to assess intra-cancer (e.g., colon transcriptome and proteome) conservation between a gene's transcript and protein, and evaluated the relationships between cancer types based on the Spearman's correlation between the inteGREAT intra-cancer integration results. The tumors originating from colon and rectal tissues exhibited strong molecular similarities (Figure S3A). Commonalities between colon and rectal samples were previously noted (Hoadley et al., 2014). Our results expanded those findings through the use of only two platforms, one not included in (Hoadley et al., 2014). Breast cancer subtypes classified as luminal A and B previously based on their transcriptome signature (Lehmann et al., 2011) were also significantly correlated (Figure S3A). These observations corroborate with previous work (Lehmann et al., 2011), where similarity between mutation, copy number, and DNA methylation of these breast cancer subtypes were reported.

**Figure 4:**
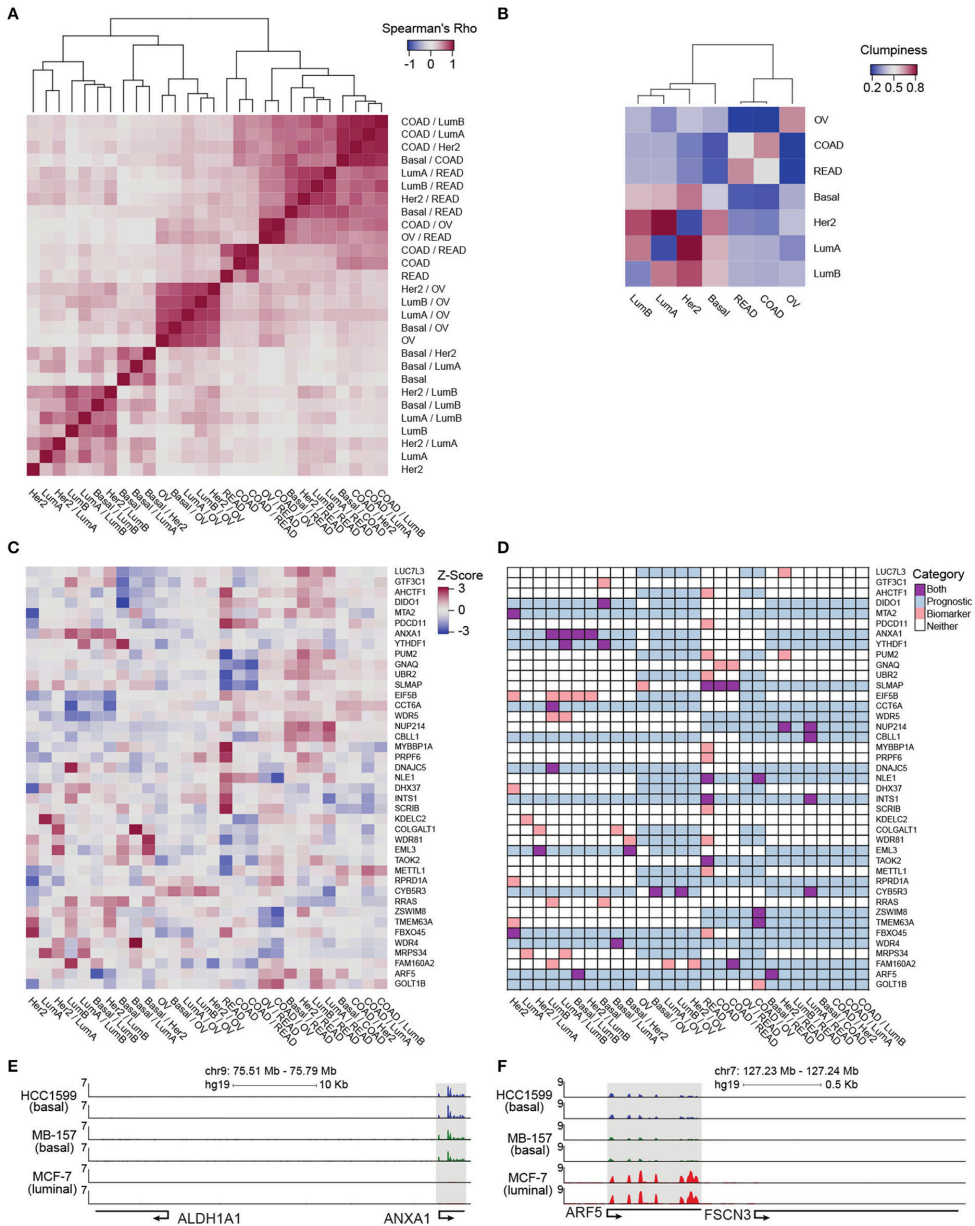
Pan-cancer differential integration. **(A,B)** Elucidate relationships between the tumor molecular features and tissue-of-origin. **(A)** Heatmap of Spearman correlations between local similarities of differential integrations, where each tile is the Spearman correlation between two differential integration similarity score vectors; **(B)** Heatmap of clumpiness values of cancer types from the dendrogram of hierarchically clustered columns of Spearman's rhos for the differential integrations in **(A)**. **(C)** Identification of putative diagnostic biomarkers. Heatmap of genes with at least one outlier in a differential integration analysis. Columns underwent *z*-score normalization before outlier removal, rows after removal. Genes with CI widths >0.04 were removed. **(D)** Prognostic significance of putative biomarkers from **(C)** inferred from survival analysis of clinical outcomes reported in the Pathology Atlas database. Each gene was designated as one of four states in each comparative study: an outlier comparison for that gene (orange), significant prognosis of that gene in a tissue in that comparison (blue), both an outlier and prognostic (purple), and neither (white). RNA-seq analysis of *ANXA1* **(E)** and *ARF5* **(F)** expression in HC1599 (basal), MB-157 (basal), and MCF-7 (luminal) cell lines.

To provide a more refined and quantitative view of relationships among the tumor types included in our analysis, we complemented the intra-cancer integration analysis with inter-cancer integration analysis by applying inteGREAT to each cancer pair (e.g., colon vs. ovarian transcriptome and proteome data sets). Specifically, the relationship between two inter-cancer integration analyses was represented by the Spearman correlation between their gene product similarity score vectors. All gene products were included to provide an unbiased matrix containing the relationships between integration comparisons. Hierarchical clustering of the intra-cancer and differential integration identified seven distinct clusters (five branches as cut distance 1.54, one of which consists of three branches at cut distance 1.33) and yielded a distinct relationship between their transcript/protein expressions and tissues of origin (Figure 4A). We observed that the BRCA luminal A/B subtypes clustered together. The BRCA basal subtypes were distinct from the luminal subtypes, an observation that was noted earlier by integrative genomics analysis (Hoadley et al., 2014). Ovarian tumors form a distinct cluster which exhibited their differences from BRCA subtypes. Colon and rectal cancers were distinctly identifiable and neighbored the cluster consisting of differences between the ovarian and colorectal tumors. The last two clusters were formed by the differences between the rectal and colon versus breast tumors (Figure 4A). Interestingly, the HER2-positive breast cancer subtype was spread across the dendrogram (Figure 4A). In stark contrast, ovarian cancer was strongly segregated in a single subtree, only appearing elsewhere close to colon and rectal cancers, corroborating the earlier findings (Hoadley et al., 2014).

In order to clarify the aggregation of ovarian cancer comparisons and the promiscuous placements of HER2-positive breast cancer subtype within the dendrogram, we applied a clumpiness measure (Schwartz et al., 2016; Meng et al., 2017) to the tree in Figure 4A (see Supplementary Materials). Clumpiness is a measure of aggregation of labels within a hierarchical structure. With this measure, we can quantify the degree of dispersion of a cancer throughout the dendrogram. In contrast to a previous pan-cancer analysis using a single platform (Lu et al., 2005), we observed that ovarian cancer was indeed the least similar to all other cancer types included in our pan-cancer analysis, but shared a stronger relationship with colorectal cancers than the breast cancer subtypes (Figure 4B heatmap). Interestingly, while the colon and rectal cancers were aggregated with themselves, similar to ovarian cancers, the breast cancer subtypes were not aggregated into a single group (Figure 4B heatmap). In fact, HER2-positive and luminal A subtypes had low clumpiness values with themselves, meaning their comparisons were scattered across the entire dendrogram of Figure 4A (Figure 4B heatmap). This finding implies a weak intra-cancer relationship; these tumor types have stronger similarities with other types than their own. Furthermore, by hierarchically clustering these clumpiness values, we observed an overall relationship of cancers (Figure 4B). The dendrogram consists of two distinct groups: the breast cancer subtypes and the colorectal/ovarian subtypes. We found that luminal A and B were the most related and as a sub-group the most different from basal subtype. This observation demonstrates the discrepancy between the luminal and basal cells in the mammary ducts, in line with previous studies (Farmer et al., 2005). Furthermore, we observed that the HER2-positive subtype was more closely related to luminal than basal subtypes (Figure 4B), possibly because some of the luminal B tumors carry ERBB2 amplifications, while all the tumors classified as basal subtype in our data set are triple negative and lack HER2 expression. We also observed that colon and rectal cancers converged into a colorectal cancer type (Figure 4B), as reported earlier in (Hoadley et al., 2014). This finding reflects the close tissue proximity of the two cancers. Most dissimilar to all other cancer types in our data set was ovarian cancer (Figure 4B), which is known to have a unique signature (Li Y. et al., 2017). Although the tissue-of-origin as expected is the dominant driver of cancer types segregation (Lu et al., 2005), our integrative analysis using inteGREAT rediscovered exceptions by demonstrating the relationships between the major breast cancer subtypes using measurements from two platforms instead of five (Hoadley et al., 2014) (Figure 4B). Together, these data demonstrate the inteGREAT accuracy when analyzing real biological data sets.

### 3.4. Pan-cancer differential integration identifies putative prognostic biomarkers

In order to explore the genes acting as possible prognostic biomarkers for each cancer type, we first identified subtype-specific putative biomarker genes. We considered a gene as a putative biomarker if its normalized cosine similarity distribution, generated from the collection of inteGREAT intra- and inter-cancer integration analyses, had at least one outlier value, defined as 1.5 times the interquartile range plus or minus the upper and lower quartile, respectively (see Supplementary Materials). An outlier represents a gene that was scored significantly different in one integration analysis compared to the others.

Then we assessed the clinical relevance of these putative biomarker genes. We mined the Pathology Atlas (Uhlen et al., 2015) and examined how the expression of our nominated putative biomarker genes correlated with the clinical outcomes as measured by the significance in the differential overall patient survival times for each specific malignancy included in our pan-cancer data set (see Supplementary Materials). The intra-cancer integration analysis identified 93 putative biomarker genes (Figure S3B). The expression level of 38 out of 93 putative biomarkers identified by intra-cancer integration (40.9%) significantly correlated with the differential overall survival rate of cancer patients (Figure S3C). When a similar analysis was performed considering both intra- and inter-cancer inteGREAT analysis, the number of putative biomarkers were reduced to 41 (Figure 4C), 20 of which (48.8%) exhibited significant correlation with differential overall survival rate (Figure 4D). Together, we observed that differential integration improved the rate of putative biomarker identification by 8%. This observation underscores the importance of differential integration analyses and suggests that finding how much a gene product is conserved within a tumor type but differs from other tumor types can facilitate discovery of clinically relevant biomarkers.

Earlier studies elucidate the significance of a number of biomarkers nominated by inteGREAT to the pathobiology of their corresponding disease. For example, *CBLL1*, or *HAKAI*, is a proto-oncogene implicated in colorectal cancers (Zhou et al., 2011). *MYBBP1A* is known to bind and activate p53 and is involved in colorectal cancers (Kuroda et al., 2011; Ono et al., 2013; Kumazawa et al., 2015; Li X. L. et al., 2017). Our predicted ovarian specific biomarker *CYB5R3* is reported to be involved in ovarian cancer (Yamanoi et al., 2016). Together, our analysis suggests that integration of differential transcriptome and protein data sets improves the specificity of biomarker identification.

Using inteGREAT, we also identified *ANXA1* and *ARF5* to be putative biomarkers for basal and luminal breast cancer subtypes with potential prognostic significance. High expression of *ANXA1* promotes metastasis of basal-like tumors and associates with poor prognosis in this breast cancer subtype (de Graauw et al., 2010; Bhardwaj et al., 2015). To verify *ANXA1* as a biomarker for basal vs. luminal subtypes, we performed RNA-seq to measure transcripts of three breast cancer cell lines: HCC1599 (basal), MB-157 (basal), and MCF-7 (luminal). As predicted by the inteGREAT pan-cancer analysis, *ANXA1* exhibited significantly higher expression in the two basal cell lines HCC1599 and MB-157 (Figure 4E). Furthermore, it has been previously shown that *ANXA1* has lower expression in luminal than basal tumor types (de Graauw et al., 2010), confirming its identification as a biomarker by inteGREAT (de Graauw et al., 2010). Conversely, our RNA-seq experiments in breast cancer cell lines confirmed that *ARF5* is highly expressed in MCF-7 luminal cells, but not expressed in basal cell lines (Figure 4F). These data, together with our pan-cancer analysis, propose *ARF5* as a possible biomarker of luminal breast cancer subtype which has a tumor subtype-specific gene-program in transcript and protein with potential prognostic significance.

## 4. Discussion

High-throughput assays have enabled global profiling of different aspects of tumor characteristics, from the transcriptome to the proteome. A significant step toward more effective cancer treatment is to leverage diverse genome-scale data sources to complement investigation of tumor characteristics. Despite the recent advances in proteomic technologies, further reproducibility and quality control procedures should be developed (Tabb, 2013; Mertins et al., 2016; Bittremieux et al., 2017). Nevertheless, the holistic and integrated views of cancer could facilitate discovery of molecular-based diagnostic and prognostic biomarkers and guide precise clinical management and therapeutic decision-making. While recent algorithms attempt to integrate data sources for individual tumor types, there are still unmet needs for analytic approaches to enable differential integration analyses to facilitate the discovery of tumor-specific biomarkers from an integrative view of tumor biology. Here, we have presented inteGREAT, an algorithm to integrate transcript and protein abundance data and detect differential biomarkers between multiple cancer subtypes.

We have shown the robustness of inteGREAT using simulations controlling for multiple sources of biological noise. In addition, we demonstrated the accuracy and utility of inteGREAT to infer differences and similarities of four tumor types. inteGREAT confidently identified previously published diagnostic biomarkers of basal and luminal breast cancer subtypes from their respective transcriptomic and proteomic data. Using a measure of clumpiness for summarizing hierarchical trees, inteGREAT performed differential integration for seven different cancer subtypes and detected convergence and divergence of tumors from various tissues-of-origin according to their transcriptomic and proteomic characteristics. Furthermore, inteGREAT identified putative biomarkers for each subtype with potential prognostic significance.

Using multiple analyses, we demonstrated that integration of transcriptome and protein interactomes enhances reliability of biomarker discovery rather than using only each of these measurements alone. We propose that measuring biological systems from more than one perspective diminishes the effect of missing data and noisy assays, while simultaneously elucidating new relationships between disparate data sources that cannot be captured in a single assay.

inteGREAT is a generic algorithm for measuring inter-network similarity and is able to report differential information. While in this study we only used inteGREAT for biomarker detection from transcriptome and proteome data in different cancer subtypes, our flexible implementation of inteGREAT enables new analysis of networks from a variety of biological sources, including the epigenome, CNVs, and mutation data. This algorithm is a powerful tool to further cancer biomarker discovery to aid in therapeutics advancements.

## Data availability statement

The data sets analyzed for this study can be found in The Cancer Genome Atlas (TCGA) https://portal.gdc.cancer.gov/, the Clinical Proteomic Tumor Analysis Consortium (CPTAC) https://cptac-data-portal.georgetown.edu/cptacPublic/, and the Pathology Atlas https://www.proteinatlas.org/pathology.

## Author contributions

RF and GS designed experiments. GS and RF designed the algorithm. GS implemented the software, collected, and organized data. JP and YZ performed and analyzed sequencing experiments. RF and GS wrote the manuscript with comments from all authors. RF conceived the project, administrated the experiments and analyses, and provided expert advice.

### Conflict of interest statement

The authors declare that the research was conducted in the absence of any commercial or financial relationships that could be construed as a potential conflict of interest.
