# Supplementary material for: Differential Integration of Transcriptome and Proteome Identifies Pan-Cancer Prognostic Biomarkers
Source: Front Genet. 2018 Jun 15;9:205. doi: 10.3389/fgene.2018.00205 (PMC6018483; doi:10.3389/fgene.2018.00205)
Supplement: Supplementary file 1 [file Image_1.pdf]

Supplementary Material: Differential integration of transcriptome and proteome identifies pan-cancer prognostic biomarkers

1 SUPPLEMENTARY MATERIAL AND METHODS

1.1 Simulations

`inteGREAT` uses either global or local measures of networks similarity to detect changes in the neighborhoods of each gene. In order to assess the performance of these network similarities for use between networks in biological systems, we applied `inteGREAT` on synthetic networks. Using our program `random-level-network`, we can generate our own random networks and capture the changes.

1.1.1 Synthetic network creation

`random-level-network` generates a base network using the Barabási-Albert model (power law structure) (Barabási and Albert, 1999) to simulate a biological network and randomly chooses a certain number of vertices to permute the network edges, while maintaining the degree of those vertices. Noise is injected in several ways:

- Initial network is random based off of the Barabási-Albert model.
- Initial network edges are randomly chosen from a standard normal distribution.
- Random permutation of edges of random vertices in the other data sources. These vertices are the basis of the simulation – we would expect these vertices to be ranked highly in the ranked-order list for vertex similarity.
- Random deletion of vertices.
- Random injection of noise. We add to each edge a random value chosen from a normal distribution of mean 0 and varying standard deviation, depending on the level of noise.

1.1.2 Accuracy measure

Generating a set of synthetic data sources, we create a set of m tuples containing the vertex id and the vertex similarity scores, $X = \{(v_1, c_1), (v_2, c_2), \ldots, (v_m, c_m)\}$, and a set of known changed vertices, Y. We rank order the scores, such that the lowest score is 1 and the highest score is m, resulting in the sorted set $R = \{(v_1, r_1), (v_2, r_2), \ldots, (v_m, r_m)\}$.

If the algorithm was able to detect all changed vertices perfectly, then the vertex similarity scores for all $v \in Y$ should be the lowest values in R. If the algorithm was perfectly wrong, then the vertices in Y should have the highest correspondence in R. Lastly, if the algorithm randomly assigned node correspondence scores, then the vertices in Y should be uniformly distributed in R. In order to measure the accuracy without a binary perfect or imperfect value, then we need to take into account the overall distance of all $v \in Y$ from the end of R.

We test accuracy by a measure using distances based on rank: we sum the distance from each vertex in Y whose score is not in the top ranking ($r \in [1, |Y|]$), then divide by the number of genes not in the top ($[1, |Y|]$) or bottom ($[|R| - |Y|, |R|]$) rank. More specifically, we generate a new set $R' = \{(v, r - |Y|) \mid (v, r) \in R \wedge v \in Y \wedge r - |Y| > 0\}$ containing the "imperfect" vertices and how far they are from the top of the ranked-order list. Then our accuracy is calculated as

$$1 - \frac{\sum R'}{\sum_{i=|R|-2|Y|}^{|R|-|Y|} i}.$$

In summary,

1. Order the tuples by their vertex similarity scores.
2. Rank transform the scores such that 1 is the lowest vertex similarity score.
3. Subtract the number of known changed vertices from all rankings.
4. Remove rankings that are less than 1 (so we essentially get a ranking **below** the top few vertices).
5. Get all known changed vertices from this ranking that are not in the top few, so their ranking will be greater than 0.
6. Add up those ranks and divide by the theoretical worst case, the sum of the "$|Y|$th" lowest rankings.

7. Subtract this value from 1.

The defined accuracy measure ranges from 0 to 1 for the worst performing to the best performing respectively and takes into account the total distance of the known changed vertices from the top ranking positions. We would expect uniformly random placement to be approximately 0.5.

1.1.3 Range of tests

We applied `inteGREAT` to random networks generated with `random-level-network` with ten runs for each variation (Table S1). 5% of vertices were permuted. Those vertices were expected to be near the top ranking.

1.2 Biological data collection

Transcriptome and proteome samples from serous ovarian carcinoma (OV) (Bell et al., 2011; Zhang et al., 2016), breast cancers (BRCA) (Koboldt et al., 2012; Mertins et al., 2016), colon (COAD) and rectal (READ) adenocarcinomas (Muzny et al., 2012; Zhang et al., 2014) were obtained from TCGA (Grossman et al., 2016). Transcriptome data was obtained using the GDC Data Transfer Tool `gdc-client` (https://gdc.cancer.gov/access-data/gdc-data-transfer-tool). Proteome data was collected from the CPTAC Data Portal (https://cptac-data-portal.georgetown.edu/cptacPublic/). Each sample was upper quartile normalized and $\log_2$ transformed. Any genes that appeared in less than five samples in the upper quartile were removed.

1.2.1 mRNA expression measurement

RNA-seq Cells were washed once with 1x PBS before resuspending pellet in 350 µl Buffer RLT Plus (Qiagen) with 10% 2-Mercaptoethanol (Sigma), vortexed briefly, snap-frozen on dry ice, and stored at −80 °C. Subsequently, total RNA was isolated using the RNeasy Plus Micro Kit (Qiagen). RNA integrity numbers were determined using a TapeStation 2200 (Agilent), and all samples used for RNA-seq library preparation had RIN numbers greater than 9.5. Libraries were prepared using the SMARTer® High-Input Strand-Specific Total RNA-seq for Illumina kit (Clontech). Libraries were single-end sequenced (75 bp) on a NextSeq 550. Three biological replicates were performed for each cell line.

RNA-seq data analysis For HCC-1599, MB-157 RNA-Seq reads were aligned to hg19 using STAR (Dobin et al., 2012). MCF-7 data (Dunham et al., 2012) were downloaded from the Short Read Archive (SRA) (`https://www.ncbi.`

nlm.nih.gov/sra) and reprocessed with the same methodology. RNA-seq display files were generated using a combination of UCSC (Kent et al., 2002) and BAM tools (Barnett et al., 2011) and visualized with the UCSC Genome Browser. Scaling for all RNA-Seq tracks in figures is equal to local paired-end fragment coverage x (1,000,000 / totalCount).

1.3 Basal vs. luminal integration

While generating synthetic networks results in a well-controlled environment, real data was used to test inteGREAT in a biological context. Known biomarkers for basal vs. luminal breast cancer subtypes exists, so inteGREAT was applied to the transcriptome and proteome samples of basal and luminal subtypes using global or local similarity methods. Integration was applied in three ways, representing three different possibilities for this analysis: differential integration of basal vs. luminal with the transcriptome and the proteome, and ”without” integration using network similarity of basal vs. luminal at the transcriptome level and basal vs. luminal at the proteome level. The analyses that did not use both the transcriptome and proteome were expected to perform worse than the analysis that used both.

1. inteGREAT was applied such that the basal and luminal samples were concatenated and the transcriptome and proteome were data sources. In this scenario, two correlation networks were created: one from the basal and luminal transcriptome samples and one from the basal and luminal proteome samples. Network similarity was then used between the two networks.
2. inteGREAT was applied such that the basal transcriptome and luminal transcriptome were treated as two data sources. In this scenario, two correlation networks were created: one from the basal transcriptome samples and one from the luminal transcriptome samples. Network similarity was then used between the two networks.
3. inteGREAT was applied such that the basal proteome and luminal proteome were treated as two data sources. Same as analysis 2. but using the proteome instead of the transcriptome.

Pre-ranked gene set enrichment analysis (GSEA) was applied to the three resulting rankings of genes with default options to obtain enriched pathways.

The transcriptome and proteome rankings generated by standard fold change were calculated by subtracting the luminal upper quartile normalized and $\log_2$ transformed luminal values from basal values. The two ranked lists ranged from negative to positive values. The rankings of *ESR1* and *GATA3* were determined by their highest ranking in relation to the most negative or most positive values.

In order to control for the possibility of bias from having too many samples in one data source over another, `inteGREAT` was also applied to ten randomly sub-sampled data sets such that the number of samples in the proteome equaled that of the transcriptome. Runs were combined using rank product with 1000 permutations for the permutation test.

1.4 Pan-cancer integration

1.4.1 Clumpiness

`inteGREAT` was applied to all pairwise comparisons of basal, Her2, luminal A, luminal B, colon, rectal, and ovarian cancers. Like-to-like (intra-cancer) comparisons, such as basal to basal, were interpreted as a basic integration of the transcriptome and proteome with no differential aspect, resulting in 28 overall rankings. Genes with CI widths > 0.04 were removed. The correlation matrices were generated by using Spearman's correlation coefficient on each pairwise ranking comparison. Trees resulting from hierarchical clustering were calculated using complete linkage. In order to find the aggregation of labels in these trees, a clumpiness measure was applied to the dendrograms (Schwartz et al., 2016; Meng et al., 2017). Clumpiness defined in (Schwartz et al., 2016) analyzes the structure of any hierarchical structure to determine how aggregated or "clumped" labels within vertices are.

Briefly, for a rooted tree with the set of non-leaf and non-root vertices I, leaf vertices T whose parents are in I, and labels of the leaves $L = \{L_1, L_2\}$, the clumpiness between L_1 and L_2 is defined as

$$C(L) = \frac{1}{2}\left(\prod_{i=1}^{2} \frac{x}{y_i}\right)^{1/2}.$$

Here, x is the number of vertices in I that contain at least one vertex of each label in its descendant leaves weighed by the shortest paths to those leaves, while

$y_i = \frac{|L_i|}{|T|}$, the fraction of leaves belonging to the label L_i. For a more general and descriptive definition of clumpiness, see (Schwartz et al., 2016).

For the gene heatmaps, where the rows were genes and the columns were integration analyses, each column was z-score normalized, then genes with at least one column outlier were gathered. Finally, for visualization, rows were z-score normalized.

1.4.2 Biomarker identification

In order to detect genes that were relevant in certain cancer types rather than others, disease comparisons that appeared as outliers for genes were interpreted as specific to those diseases, or ”putative biomarkers”. For both the seven integration cancer type analysis (intra-cancer) and the 28 differential integration analysis (inter-cancer) the following process was applied to each analysis separately. Genes with CI widths > 0.04 were removed and rankings were z-score normalized. Outliers were designated by finding values $1.5 *$ interquartile range smaller or larger than the lower and upper quartiles, respectively. These outlier analysis were considered diseases of interest for that specific gene.

1.4.3 Linking biomarkers with overall survival

With each gene having a designated set of diseases of interest, the relevance of that biomarker was determined using overall survival. Log-rank p-values from Kaplan-Meier curves of overall survival were obtained from the Pathology Atlas, part of the Human Protein Atlas (Uhlen et al., 2015). For each cancer type, we considered a biomarker as being correlated with overall survival if a log-rank test resulted in $p < 0.05$.

2 SUPPLEMENTARY TABLES AND FIGURES

2.1 Figures

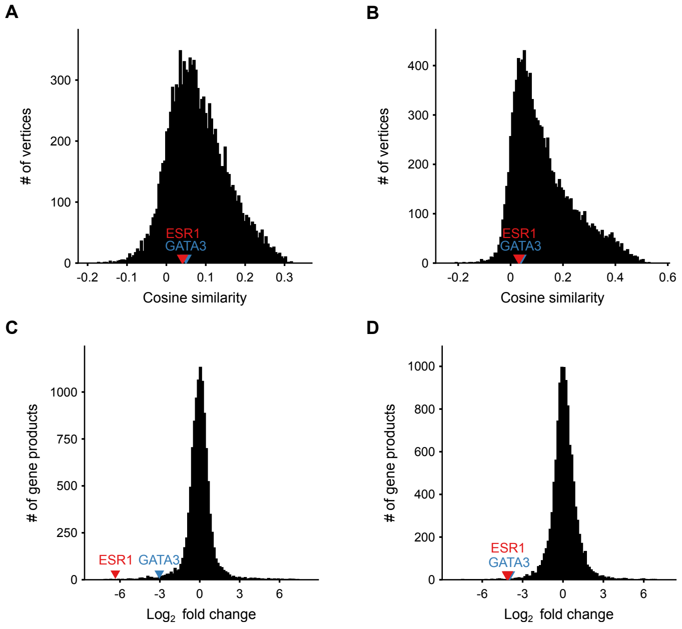

Figure S1. Related to Figure 3. Detection of *ESR1* and *GATA3* from differential of basal and luminal subtypes without integration of multiple data sources. *ESR1* and *GATA3* are marked with red and blue respectively. (A) Local similarity using only the transcriptome. (B) Local similarity using only the proteome. (C) $\log_2$ fold change using only the transcriptome. (D) log_2 fold change using only the proteome.

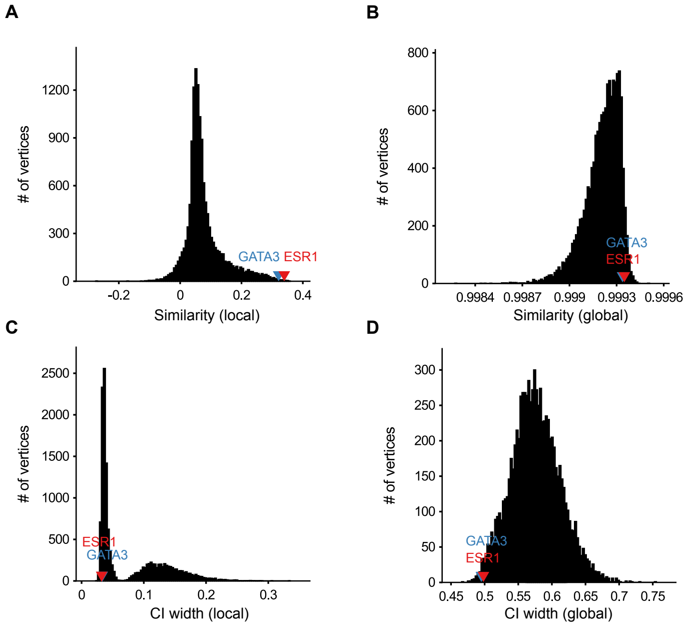

Figure S2. Related to Figure 3. Detection of *ESR1* and *GATA3* using ten runs of subsampling differential integration of basal vs. luminal. *ESR1* and *GATA3* are marked with red and blue respectively. (A) A sample run of differential integration using local similarity of basal vs. luminal. (B) A sample run using global similarity. (C) A sample run using local similarity showing the confidence interval width. (D) A sample run using global similarity showing the confidence interval width.

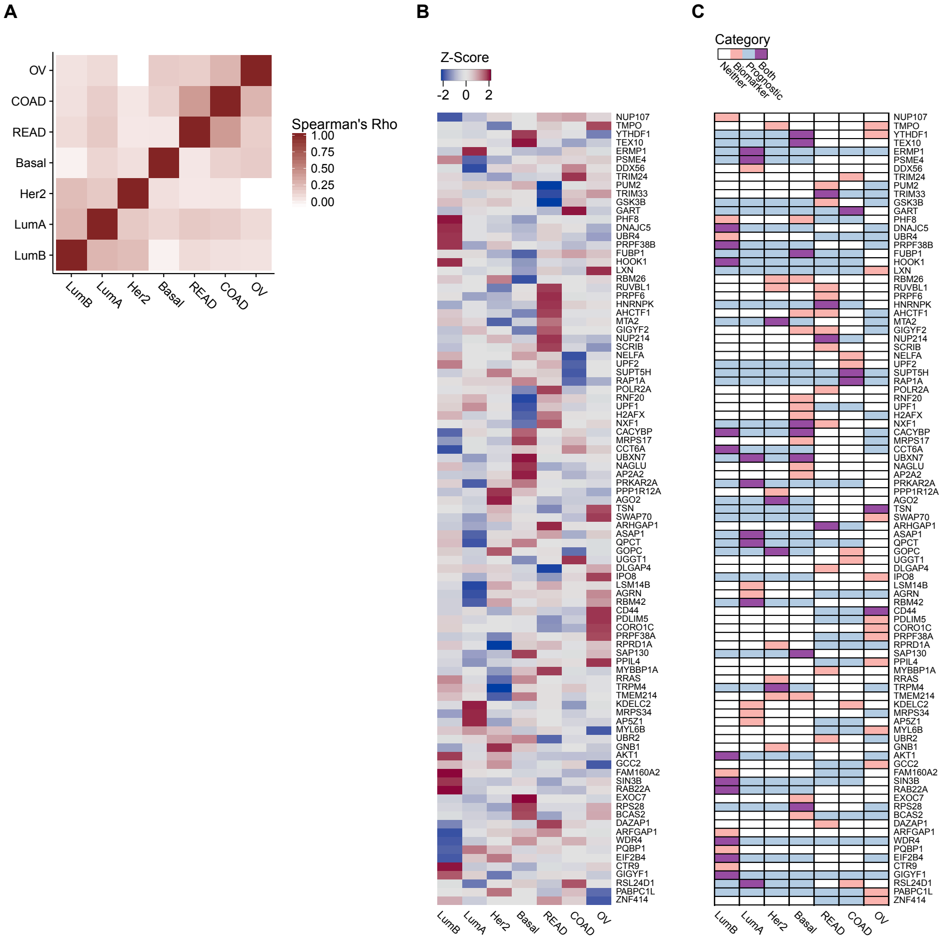

Figure S3. Related to Figure 4. Intra-cancer integration using the transcriptome and proteome with local similarity. (A) Heatmap of Spearman correlations between local similarities of basic transcriptome and proteome integration for each cancer type. (B) Heatmap of genes with outlier values in at least one integration analysis. Local similarities of all genes for each integration were z-score normalized before outlier removal, then the values for all integration analyses for each gene were z-score normalized after removal. Genes with CI widths > 0.04 were removed. (C) Putative prognostic biomarker detection from (B). There are four states for each cell: the outlier comparison for that gene as a putative biomarker (orange), significant prognosis of that gene in a tissue in that comparison (blue), both a putative biomarker and prognostic (purple), and neither (white).

2.2 Tables

Table S1. Variation of parameters in simulations.

Property		Minimum	Maximum	Interval
Vertices to recover	(%)	5	5	0
Data sources	(#)	2	3	1
Network size	(# vertices)	200	1000	200
Edges permuted	(%)	0	50	10
Vertices deleted	(%)	0	50	10
Noise injection	(σ)	0	6	1

Table S2. Numbers of samples for each cancer type.

Type	Transcriptome	Proteome
Basal	10	18
Her2	5	12
Luminal A	11	23
Luminal B	15	24
COAD	59	64
READ	30	31
OV	119	206

Table S3. Top 20 GSEA pathways enriched with the highest ranked gene IDs for the differential integration of the transcriptome and proteome from basal vs. luminal samples.

NAME	SIZE	ES	NES	NOM	FDR	FWER
SCHUETZ_BREAST_CANCER_DUCTAL_INVASIVE_UP	318	0.422	8.44	0	0	0
FARMER_BREAST_CANCER_BASAL_VS_LULMINAL	306	0.354	7.15	0	0	0
VANTVEER_BREAST_CANCER_ESR1_UP	160	0.439	6.43	0	0	0
ANASTASSIOU_CANCER_MESENCHYMAL_TRANSITION_SIGNATURE	62	0.667	6.35	0	0	0
CHICAS_RB1_TARGETS_CONFLUENT	481	0.249	6.29	0	0	0
DUTERTRE_ESTRADIOL_RESPONSE_24HR_UP	293	0.306	5.96	0	0	0
GRAHAM_CML_DIVIDING_VS_NORMAL_QUIESCENT_UP	171	0.382	5.83	0	0	0
PICCALUGA_ANGIOIMMUNOBLASTIC_LYMPHOMA_UP	187	0.367	5.80	0	0	0
CHARAFE_BREAST_CANCER_LUMINAL_VS_MESENCHYMAL_DN	397	0.251	5.68	0	0	0
SENGUPTA_NASOPHARYNGEAL_CARCINOMA_UP	253	0.305	5.57	0	0	0
NABA_CORE_MATRISOME	201	0.338	5.51	0	0	0
RODWELL_AGING_KIDNEY_UP	415	0.236	5.47	0	0	0
BERENJENO_TRANSFORMED_BY_RHOA_UP	487	0.215	5.46	0	0	0
SMID_BREAST_CANCER_LUMINAL_B_DN	451	0.224	5.40	0	0	0
LINDGREN_BLADDER_CANCER_CLUSTER_2B	343	0.255	5.39	0	0	0
SMID_BREAST_CANCER_NORMAL_LIKE_UP	356	0.241	5.34	0	0	0
KOBAYASHI_EGFR_SIGNALING_24HR_DN	230	0.307	5.33	0	0	0
CHARAFE_BREAST_CANCER_LUMINAL_VS_BASAL_DN	400	0.233	5.31	0	0	0
DOANE_BREAST_CANCER_ESR1_UP	101	0.455	5.31	0	0	0
LIU_PROSTATE_CANCER_DN	388	0.229	5.20	0	0	0

Table S4. Top 20 GSEA pathways enriched with the lowest ranked gene IDs for the differential integration of the transcriptome and proteome from basal vs. luminal samples.

NAME	SIZE	ES	NES	NOM	FDR	FWER
BIOCARTA_PROTEASOME_PATHWAY	28	−0.567	−3.64	0	0	0
KEGG_PROTEASOME	42	−0.391	−2.99	0	0.001 34	0.005 00
REACTOME_CELL_CYCLE	358	−0.136	−2.98	0	0.000 893	0.005 00
REACTOME_HIV_INFECTION	185	−0.185	−2.91	0	0.001 33	0.0100
REACTOME_MITOTIC_G1_G1_S_PHASES	125	−0.218	−2.88	0	0.001 28	0.0120
REACTOME_AUTODEGRADATION_OF_CDH1_BY_CDH1_APC_C	55	−0.327	−2.85	0	0.001 33	0.0150
REACTOME_AUTODEGRADATION_OF_THE_E3_UBIQUITIN_LIGASE_COP1	46	−0.354	−2.84	0	0.001 37	0.0180
REACTOME_CDT1_ASSOCIATION_WITH_THE_CDC6_ORC_ORIGIN_COMPLEX	53	−0.331	−2.84	0	0.001 26	0.0190
REACTOME_CLASS_A1_RHODOPSIN_LIKE_RECEPTORS	72	−0.280	−2.78	0	0.001 59	0.0270
REACTOME_SIGNALING_BY_WNT	61	−0.303	−2.78	0	0.001 59	0.0300
REACTOME_G1_PHASE	33	−0.408	−2.77	0	0.001 50	0.0310
REACTOME_APC_C_CDH1_MEDIATED_DEGRADATION_OF_CDC20_AND_OTHER_APC...	62	−0.294	−2.77	0	0.001 46	0.0330
REACTOME_HOST_INTERACTIONS_OF_HIV_FACTORS	118	−0.221	−2.76	0	0.001 56	0.0380
REACTOME_M_G1_TRANSITION	75	−0.267	−2.74	0.001 95	0.001 71	0.0440
REACTOME_CDK_MEDIATED_PHOSPHORYLATION_AND_REMOVAL_OF_CDC6	45	−0.345	−2.73	0	0.001 74	0.0480
REACTOME_P53_DEPENDENT_G1_DNA_DAMAGE_RESPONSE	52	−0.322	−2.72	0	0.001 69	0.0500
REACTOME_SCF_BETA_TRCP_MEDIATED_DEGRADATION_OF_EMI1	48	−0.325	−2.71	0	0.001 82	0.0570
REACTOME_PACKAGING_OF_TELOMERE_ENDS	27	−0.438	−2.70	0	0.001 83	0.0610
REACTOME_VIF_MEDIATED_DEGRADATION_OF_APOBEC3G	48	−0.329	−2.68	0	0.002 38	0.0830

Table S5. Top 20 GSEA pathways enriched with the highest ranked gene IDs for the differential analysis of the transcriptome from basal vs. luminal samples.

NAME	SIZE	ES	NES	NOM	FDR	FWER
SCHUETZ_BREAST_CANCER_DUCTAL_INVASIVE_UP	289	0.469	9.20	0	0	0
SOTIRIOU_BREAST_CANCER_GRADE_1_VS_3_UP	138	0.618	8.26	0	0	0
POOLA_INVASIVE_BREAST_CANCER_UP	213	0.474	8.16	0	0	0
ROSTY_CERVICAL_CANCER_PROLIFERATION_CLUSTER	128	0.596	7.89	0	0	0
WALLACE_PROSTATE_CANCER_RACE_UP	218	0.435	7.42	0	0	0
KOBAYASHI_EGFR_SIGNALING_24HR_DN	222	0.417	7.20	0	0	0
GOBERT_OLIGODENDROCYTE_DIFFERENTIATION_UP	452	0.292	7.11	0	0	0
DUTERTRE_ESTRADIOL_RESPONSE_24HR_UP	265	0.365	6.89	0	0	0
SMID_BREAST_CANCER_NORMAL_LIKE_UP	275	0.355	6.62	0	0	0
ANASTASSIOU_CANCER_MESENCHYMAL_TRANSITION_SIGNATURE	61	0.678	6.22	0	0	0
WHITEFORD_PEDIATRIC_CANCER_MARKERS	101	0.526	6.10	0	0	0
CROONQUIST_IL6_DEPRIVATION_DN	94	0.538	5.98	0	0	0
PUJANA_BRCA2_PCC_NETWORK	361	0.271	5.94	0	0	0
SHEDDEN_LUNG_CANCER_POOR_SURVIVAL_A6	374	0.266	5.88	0	0	0
REACTOME_DNA_REPLICATION	168	0.387	5.87	0	0	0
REACTOME_MITOTIC_M_M_G1_PHASES	151	0.393	5.80	0	0	0
GRAHAM_CML_DIVIDING_VS_NORMAL_QUIESCENT_UP	157	0.397	5.75	0	0	0
GRAHAM_NORMAL_QUIESCENT_VS_NORMAL_DIVIDING_DN	81	0.533	5.72	0	0	0
CHANG_CYCLING_GENES	127	0.437	5.69	0	0	0
ZHANG_TLX_TARGETS_60HR_DN	240	0.317	5.64	0	0	0

Table S6. Top 20 GSEA pathways enriched with the highest ranked gene IDs for the differential analysis of the proteome from basal vs. luminal samples.

NAME	SIZE	ES	NES	NOM	FDR	FWER
SCHUETZ_BREAST_CANCER_DUCTAL_INVASIVE_UP	260	0.444	8.23	0	0	0
SMID_BREAST_CANCER_NORMAL_LIKE_UP	285	0.325	6.29	0	0	0
LINDGREN_BLADDER_CANCER_CLUSTER_2B	274	0.322	6.10	0	0	0
HSIAO_LIVER_SPECIFIC_GENES	189	0.355	5.63	0	0	0
HSIAO_HOUSEKEEPING_GENES	351	0.262	5.59	0	0	0
KEGG_COMPLEMENT_AND_COAGULATION_CASCADES	63	0.596	5.52	0	0	0
BOQUEST_STEM_CELL_UP	203	0.334	5.48	0	0	0
ANASTASSIOU_CANCER_MESENCHYMAL_TRANSITION_SIGNATURE	56	0.607	5.31	0	0	0
NABA_CORE_MATRISOME	186	0.346	5.30	0	0	0
RODWELL_AGING_KIDNEY_UP	338	0.252	5.22	0	0	0
REN_ALVEOLAR_RHABDOMYOSARCOMA_DN	351	0.240	5.10	0	0	0
PICCALUGA_ANGIOIMMUNOBLASTIC_LYMPHOMA_UP	161	0.341	5.06	0	0	0
KEGG_SYSTEMIC_LUPUS_ERYTHEMATOSUS	69	0.497	4.91	0	0	0
WALLACE_PROSTATE_CANCER_RACE_UP	181	0.300	4.73	0	0	0
IGLESIAS_E2F_TARGETS_UP	130	0.357	4.61	0	0	0
NAKAYAMA_SOFT_TISSUE_TUMORS_PCA1_UP	61	0.486	4.49	0	0	0
NABA_ECM_GLYCOPROTEINS	131	0.339	4.46	0	0	0
REACTOME_SMOOTH_MUSCLE_CONTRACTION	21	0.801	4.39	0	0	0
MCLACHLAN_DENTAL_CARIES_UP	170	0.275	4.28	0	0	0
CHICAS_RB1_TARGETS_CONFLUENT	412	0.184	4.26	0	0	0

Table S7. Rankings of *ESR1* and *GATA3* for each analysis.

Measurement	Transcriptome	Proteome	Basal	Luminal A	Luminal B	*ESR1*	*GATA3*
Fold change	•		•	•	•	5	128
Fold change		•	•	•	•	17	23
Local similarity	•		•	•	•	7641	7076
Local similarity		•	•	•	•	8898	8482
Local similarity	•	•	•			8889	2754
Local similarity	•	•		•		1688	9027
Local similarity	•	•			•	133	2345
Local similarity	•	•	•	•	•	2	7
Local similarity (10 runs)	•	•	•	•	•	2	16
Global similarity (10 runs)	•	•	•	•	•	3	22

Table S8. Top 25 ranked molecules for differential integration of basal vs. luminal using local similarity.

Molecule	Rank Product
CA12	3.95
ESR1	6.34
TBC1D9	7.57
COL1A1	7.88
WAS	8.24
HCLS1	9.16
COL5A2	10.6
TNFAIP8L2	12.0
CORO1A	12.4
FMNL1	16.2
LCP2	16.8
MLPH	19.0
COL1A2	19.4
COL5A1	21.5
TGFB1I1	22.7
GATA3	23.2
CALD1	26.1
TLE3	26.3
GLIPR2	28.1
ITGB2	31.2
KIF2C	32.9
ARMT1	34.1
IFI16	34.3
ANGPTL2	35.2
ANTXR1	37.1

Table S9. Top 25 ranked molecules for differential integration of basal vs. luminal using global similarity.

Molecule	Rank Product
MTHFD1L	4.99
TBC1D9	5.77
ESR1	6.82
CA12	6.93
RABEP1	7.33
NUDT12	10.4
GLIPR2	14.0
TLN1	20.0
RBMS1	21.3
RUNDC1	21.6
SPR	23.8
IL6ST	27.7
CBFB	28.1
TLE3	28.5
FOXA1	30.3
MTHFD2	30.8
DNMT3A	30.8
FKBP4	30.9
QKI	32.0
XPO5	32.8
FMNL2	34.3
GATA3	35.3
C12orf10	35.3
FBXO38	37.2
MLPH	39.2

REFERENCES

Barabási, A. and Albert, R. (1999). Emergence of scaling in random networks. *Science* 286, 509–512. doi:10.1126/science.286.5439.509

Barnett, D. W., Garrison, E. K., Quinlan, A. R., Stromberg, M. P., and Marth, G. T. (2011). BamTools: A c++ API and toolkit for analyzing and managing BAM files. *Bioinformatics* 27, 1691–1692. doi:10.1093/bioinformatics/btr174

Bell, D., Berchuck, A., Birrer, M., Chien, J., Cramer, D. W., Dao, F., et al. (2011). Integrated genomic analyses of ovarian carcinoma. *Nature* 474, 609–615. doi:10.1038/nature10166

Dobin, A., Davis, C. A., Schlesinger, F., Drenkow, J., Zaleski, C., Jha, S., et al. (2012). STAR: Ultrafast universal RNA-seq aligner. *Bioinformatics* 29, 15–21. doi:10.1093/bioinformatics/bts635

Dunham, I., Kundaje, A., Aldred, S. F., Collins, P. J., Davis, C. A., Doyle, F., et al. (2012). An integrated encyclopedia of DNA elements in the human genome. *Nature* 489, 57–74. doi:10.1038/nature11247

Grossman, R. L., Heath, A. P., Ferretti, V., Varmus, H. E., Lowy, D. R., Kibbe, W. A., et al. (2016). Toward a shared vision for cancer genomic data. *N. Engl. J. Med.* 375, 1109–1112. doi:10.1056/nejmp1607591

Kent, W. J., Sugnet, C. W., Furey, T. S., Roskin, K. M., Pringle, T. H., Zahler, A. M., et al. (2002). The human genome browser at UCSC. *Genome Res.* 12, 996–1006. doi:10.1101/gr.229102

Koboldt, D. C., Fulton, R. S., McLellan, M. D., Schmidt, H., Kalicki-Veizer, J., McMichael, J. F., et al. (2012). Comprehensive molecular portraits of human breast tumours. *Nature* 490, 61–70. doi:10.1038/nature11412

Meng, W., Zhang, B., Schwartz, G. W., Rosenfeld, A. M., Ren, D., Thome, J. J. C., et al. (2017). An atlas of b-cell clonal distribution in the human body. *Nat. Biotechnol.* 35, 879–884. doi:10.1038/nbt.3942

Mertins, P., Mani, D. R., Ruggles, K. V., Gillette, M. A., Clauser, K. R., Wang, P., et al. (2016). Proteogenomics connects somatic mutations to signalling in breast cancer. *Nature* 534, 55–62. doi:10.1038/nature18003

Muzny, D. M., Bainbridge, M. N., Chang, K., Dinh, H. H., Drummond, J. A., Fowler, G., et al. (2012). Comprehensive molecular characterization of human colon and rectal cancer. *Nature* 487, 330–337. doi:10.1038/nature11252

Schwartz, G. W., Shokoufandeh, A., Ontañón, S., and Hershberg, U. (2016). Using a novel clumpiness measure to unite data with metadata: Finding common sequence patterns in immune receptor germline v genes. *Pattern Recognit. Lett.* 74, 24–29. doi:10.1016/j.patrec.2016.01.011

Uhlen, M., Fagerberg, L., Hallstrom, B. M., Lindskog, C., Oksvold, P., Mardinoglu, A., et al. (2015). Tissue-based map of the human proteome. *Science* 347, 1260419–1260419. doi:10.1126/science.1260419

Zhang, B., Wang, J., Wang, X., Zhu, J., Liu, Q., Shi, Z., et al. (2014). Proteogenomic characterization of human colon and rectal cancer. *Nature* 513, 382–387. doi:10.1038/nature13438

Zhang, H., Liu, T., Zhang, Z., Payne, S. H., Zhang, B., McDermott, J. E., et al. (2016). Integrated proteogenomic characterization of human high-grade serous ovarian cancer. *Cell* 166, 755–765. doi:10.1016/j.cell.2016.05.069
